# Stem cell-derived exosomes from human exfoliated deciduous teeth promote angiogenesis in hyperglycemic-induced human umbilical vein endothelial cells

**DOI:** 10.1590/1678-7757-2022-0427

**Published:** 2023-03-15

**Authors:** Thanapat SUNARTVANICHKUL, Tawepong ARAYAPISIT, Sujiwan Seubbuk SANGKHAMANEE, Chaiyapol CHAWEEWANNAKORN, Kengo IWASAKI, Phatchanat KLAIHMON, Hathaitip SRITANAUDOMCHAI

**Affiliations:** 1 Mahidol University Faculty of Dentistry Department of Orthodontics Bangkok Thailand Mahidol University, Faculty of Dentistry, Department of Orthodontics, Bangkok, Thailand.; 2 Mahidol University Faculty of Dentistry Department of Anatomy Bangkok Thailand Mahidol University, Faculty of Dentistry, Department of Anatomy, Bangkok, Thailand.; 3 Department of Oral Medicine and Periodontology Faculty of Dentistry Mahidol University Thailand Department of Oral Medicine and Periodontology, Faculty of Dentistry, Mahidol University, Thailand.; 4 Osaka Dental University Advanced Medical Research Center, Translational Research Institute for Medical Innovation Osaka Japan Osaka Dental University, Advanced Medical Research Center, Translational Research Institute for Medical Innovation, Osaka, Japan.; 5 Mahidol University Faculty of Medicine Siriraj Hospital Siriraj Center of Excellence for Stem Cell Research Bangkok Thailand Mahidol University, Faculty of Medicine Siriraj Hospital, Siriraj Center of Excellence for Stem Cell Research, Bangkok, Thailand.; 6 Mahidol University Department of Oral Biology Faculty of Dentistry Bangkok Thailand Mahidol University, Department of Oral Biology, Faculty of Dentistry, Bangkok, Thailand.

**Keywords:** Angiogenesis, Exosome, Hyperglycemia, Mesenchymal stem cells, Guided tissue regeneration, periodontal

## Abstract

**Objective:**

To investigate the angiogenesis in human umbilical vein endothelial cells (HUVEC) under high glucose concentration, treated with exosomes derived from stem cells from human exfoliated deciduous teeth (SHED).

**Methodology:**

SHED-derived exosomes were isolated by differential centrifugation and were characterized by nanoparticle tracking analysis, transmission electron microscopy, and flow cytometric assays. We conducted *in vitro* experiments to examine the angiogenesis in HUVEC under high glucose concentration. Cell Counting Kit-8, migration assay, tube formation assay, quantitative real-time PCR, and immunostaining were performed to study the role of SHED-derived exosomes in cell proliferation, migration, and angiogenic activities.

**Results:**

The characterization confirmed SHED-derived exosomes: size ranged from 60–150 nm with a mode of 134 nm, cup-shaped morphology, and stained positively for CD9, CD63, and CD81. SHED-exosome significantly enhanced the proliferation and migration of high glucose-treated HUVEC. A significant reduction was observed in tube formation and a weak CD31 staining compared to the untreated-hyperglycemic-induced group. Interestingly, exosome treatment improved tube formation qualitatively and demonstrated a significant increase in tube formation in the covered area, total branching points, total tube length, and total loop parameters. Moreover, SHED-exosome upregulates angiogenesis-related factors, including the GATA2 gene and CD31 protein.

**Conclusions:**

Our data suggest that the use of SHED-derived exosomes potentially increases angiogenesis in HUVEC under hyperglycemic conditions, which includes increased cell proliferation, migration, tubular structures formation, GATA2 gene, and CD31 protein expression. SHED-exosome usage may provide a new treatment strategy for periodontal patients with diabetes mellitus.

## Introduction

Diabetes is a group of metabolic disorders characterized by high blood glucose levels and potentially debilitating diseases, ranked ninth among the leading causes of death worldwide.^1^ In 2019, 463 million people were diagnosed with diabetes, disregarding undiagnosed cases.^2^ The long-term complications of diabetes are associated with the destruction of blood vessels, leading to cardiovascular disease, chronic kidney disease, retinopathy, neuropathy, and periodontitis.^3^ Diabetes has a bidirectional relationship with periodontitis, especially in poorly controlled diabetes patients. The data from the US National Health and Nutrition Examination Survey (NHANES) III showed that adults with HbA1C levels higher than 9٪ had a significantly higher prevalence of severe periodontitis than those without diabetes.^4^ Periodontitis is a chronic inflammatory disease involving the destruction of the periodontal tissue, which comprises the gingiva, cementum, periodontal ligaments, and alveolar bone. Periodontal therapy aims to regenerate damaged tissues.^5^ Angiogenesis is a critical part of regenerative therapy since an established vasculature is critical for supplying nutrients, minerals, and oxygen for proper tissue development and functionality.^3 , 5^ Moreover, vascularization aids in growth factor production that helps modulate the function of various cells in periodontal tissue, such as osteoblasts, osteoclasts, and related mesenchymal stem cells (MSCs).^6^ Notably, diabetes contributes to endothelial cell dysfunction (ECD).^7^ In diabetic vasculature, the hyperglycemic condition can cause non-enzymatic glycosylation of proteins and lipids leading to the interference of normal protein function.^8^ Hyperglycemia also increases oxidative stress through several pathways. A major mechanism appears to be superoxide (O_2_^•−^) overproduction by the mitochondrial electron transport chain. Moreover, hyperglycemia can promote inflammation via induction of cytokine secretion by several cell types.^8^ Undoubtedly, the regenerative impact of ECD on angiogenesis leads to a poor response of diabetic patients to regenerative therapy. Diabetic patients had poor responses to periodontal treatment, possibly due to ECD involvement.^9^ In many situations, conventional periodontal therapy involving root surface debridement to induce healing, guided tissue regeneration, and bone graft placement cannot achieve tissue regeneration effectively. The traditional treatment for periodontitis is associated with a relatively high degree of variability in clinical outcome, and the curative effect remains unsatisfactory.^10^ As a result, the advances in generative medicine based on MSC-mediated therapies have become a more promising alternative.

Nowadays, various types of stem cells showed potential for periodontal tissue regeneration. In particular, stem cells from human exfoliated deciduous teeth (SHED), which are postnatal stem cells taken from deciduous teeth, have demonstrated potential for regeneration.^11^ SHED have the potential to differentiate into angiogenic endothelial cells and functional odontoblasts after being implanted subcutaneously into immunodeficient mice.^12^ After *in vivo* transplantation, SHED could induce bone formation, generate dentin, and survive in mouse brain along with expression of neural markers.^13 - 14^ Numerous studies also showed its ability to upregulate proangiogenic factors like vascular endothelial growth factor (VEGF).^15 - 18^ Other studies showed that conditioned medium (CM) from SHED contains its secretions, such as proteins, growth factors, and exosomes,^19^ which directly promotes angiogenesis, resulting in increased proliferation, vascular-like structure formation, and more.^20^ Another alternative is using stem cell exosomes, which are extracellular vesicles secreted by cells that can conduct cellular communication via paracrine and autocrine signaling.^21^ Compared to cell therapy, exosomes can be beneficial due to their high stability and biocompatibility, competitive prices, low cytotoxicity, and low immunogenicity.^22^ Existing researches currently support the viability of exosomes derived from SHED in promoting angiogenesis *in vivo* and *in vitro* .^23^ Especially in periodontal regeneration, the recent evidence supported the therapeutic value of SHED-derived exosomes in the regeneration of periodontal tissues using improved cell proliferation, migration, cell cycle, and osteogenic differentiation of human periodontal ligament cells.^24^ Moreover, SHED-derived exosomes also contributed to periodontal tissue regeneration by promoting neovascularization, which is a vital part of the regeneration.^25^ However, there are no studies yet to evaluate the effect of SHED-derived exosomes in hyperglycemic conditions. Therefore, our study aimed to investigate the angiogenic effects of SHED-derived exosomes (SHED-exosome) cultured in human umbilical vein endothelial cells (HUVEC) treated with high glucose concentrations. Here we looked into the possibility of using SHED-exosome as a promising treatment option for periodontal regenerative therapy in diabetic patients.

## Methodology

### Cell cultivation

This study was approved by the Ethical Committee on Human Rights Related to Human Experimentation of the Faculty of Dentistry/Faculty of Pharmacy (MU-DT/PY-IRB 2022/013.2502). SHED cells were isolated, cultured, and characterized based on our previous study.^26^ SHED was cultured in Dulbecco’s Modified Eagle Medium (DMEM, HyClone, Fisher Scientific, Loughborough, UK), with 10٪ fetal bovine serum (FBS, Biochrome, Berlin, GY), and 1٪ Penicillin-Streptomycin (Gibco, Thermo Fisher Scientific, Loughborough, UK). SHED cells at passages 4–6 were used for these experiments.

HUVEC are endothelial cells isolated from an umbilical vein of the human umbilical cord. The cells are a widely used model system to study vascular biology *in vitro* . HUVEC have been shown to be responsive to physiological and/or pathological stimuli such as high glucose, lipopolysaccharide (LPS), and shear stress.^27 - 29^ Moreover, many findings and experimental methods for evaluating vascular endothelial cell functions have been well-established for HUVEC.^30^ In this study, the HUVEC were a kind gift from Siriraj Stem cell and Advanced Vascular and Endovascular Research group, Siriraj Center of Excellence for Stem Cell Research Faculty of Medicine, Siriraj Hospital, Mahidol University. The cells were maintained in fibronectin-coated flasks and incubated using EndoGRO-VEGF Complete Culture Media Kit (endothelial cell growth medium (EGM), Merck Ltd, Darmstadt, GY). Cells were cultured at 37°C under an atmosphere of 5٪ CO_2_ and 95٪ humidity.

### Isolation and characterization of SHED-exosome

Methods for exosome isolation and characterization were performed as described previously with minor modifications.^25 , 31^ SHED was cultured until approximately 80٪ confluent, washed three times with phosphate-buffer saline (PBS), and then incubated in DMEM without FBS and antibiotics. After incubation for 48 hours, the CM was collected and centrifuged at 1,000 rpm for 5 minutes, followed by filtering through 0.2 µm filters. The CM was then concentrated in Amicon^®^ Ultra-15 10 kDa nominal molecular weight centrifugal filter at 5,000 g for 40 minutes. The exosomes were isolated using the differential centrifugation method. Following ultracentrifugation at 100,000 × g for 70 minutes at 4°C, the exosomes were resuspended in PBS and then stored at −80°C.

The concentration of exosomes was measured using Nanodrop spectrophotometers. The characteristics of the exosomes derived from SHED cells were further identified. First, the particle size distribution of exosomes was examined using Nanoparticle tracking analysis (NTA). Then, transmission electron microscopy (TEM) was used to observe the morphology of exosomes and a flow cytometer was used to detect the exosome-specific markers CD9 (Cat. 11-0098-42, Thermo Fisher Scientific), CD63 (Cat. 12-0639-42, Thermo Fisher Scientific), and CD81 (Cat. 46-0819-42, Thermo Fisher Scientific).

### Exosomes internalization

Exosomes derived from SHED cells were labeled with PHK67 green fluorescent cell linker kit (Sigma-Aldrich Corp., St. Louis, MO, USA). In brief, 1.5 μl PKH67 dye was added to 10 μg exosomes in a total of 250 μl diluent C provided in the kit and incubated at room temperature for 5 minutes. Exosomes without PKH67 staining were used as the negative control. Excessive dye was removed by centrifugation at 190,000 × g for 2 hours at 4°C. The mixture was resuspended in a complete medium and incubated with HUVEC at 37°C for 4 hours. A laser confocal microscope was used to visualize the incorporation of exosomes into HUVEC.

### Treating the HUVEC with high glucose concentration

After reaching the desired confluence, HUVEC were cultured in 5.5 mM and 25 mM of glucose, to simulate normal fasting blood glucose concentration and uncontrolled blood glucose concentration, respectively. HUVEC were adjusted according to the assigned group:

Positive control: HUVEC were cultured in 5.5 mM of glucose in an EGM.

Osmotic control: HUVEC were cultured in 25 mM of mannitol in an EGM.

Hyperglycemia-induced: HUVEC were cultured in 25 mM of glucose in an EGM.

Negative control: HUVEC were cultured in 5.5 mM of glucose in a DMEM.

All groups were cultured for four days and were then investigated for angiogenesis.

Effects of SHED-exosome on angiogenesis

#### Cell counting Kit-8 assay

The Cell Counting Kit-8 assay (CCK-8 assay, Dojindo, Kumamoto, Jp) was used to evaluate cell proliferation according to the manufacturer’s instructions. Briefly, 3×10^3^ HUVEC per well were seeded in a 96-well plate and incubated with exosomes (10 μg/ml) or EGM (control). A total of six wells were designed for each group. On days 0, 2, 4, 6, and 7, the CCK-8 working solution was added to each well and incubated for 2 hours. The optical density at 450 nm was subsequently measured using a microplate reader.

#### Migration assay

Scratch assays were used to evaluate the cell migration. Briefly, 1×10^5^ HUVEC were seeded in 24-well plates. Cells at 90٪–100٪ confluency were subjected to single vertical scratches using a 200 µL sterile pipette tip and then washed with EGM to remove detached cells. SHED-exosome (10 μg/ml) or EGM medium (control) were added, and images were recorded at 0, 6, and 12 hours after scratching using an optical microscope. The closure distance was analyzed using Image Analysis J (Olympus, Alexandra, SG). The rate of wound closure was estimated as follows:

Rate of wound closure =


$$\frac{\text{Mean initial wound width - Mean remaining width}}{\text{Mean initial wound width}}\times 100$$

#### Tube formation assay

Matrigel^®^ Matrix (60 μl, Corning, Glendale, AZ, USA) was added to pre-cooled 96-well plates and were incubated at 37°C for 1 hour. The HUVEC cells were seeded at 1.5×10^4^ cells per well into each Matrigel-coated well. The network structures in 6 hours were captured using phase contrast microscopy. Quantitative analysis was performed in WimTube image analysis (Onimagin Technologies SCA, Córdoba, SP). The analysis algorithm was based on tubule characteristics. The results used in this study were demonstrated in the total covered area, total tube length, total branching points, and the total number of loops of each experimental group.

Moreover, a confocal microscope (Leica Microsystems CMS GmbH, Mannheim, DEU) was used for live cell imaging of tube formation for 17 hours. The representative images were exported as a time-lapse movie.

## CD31 immunofluorescence staining

The network structures in 12 hours were fixed with 2٪ paraformaldehyde (Sigma-Aldrich) and 1٪ glutaraldehyde (Sigma-Aldrich). After washing three times with PBS, cells were permeabilized using 0.2٪ Tween 20 (Sigma-Aldrich) and 0.5٪ Triton X-100 (Sigma-Aldrich). After blocking with 3٪ bovine serum albumin, the cells were incubated with mouse monoclonal anti-human CD31 primary antibody (Abcam, Cambridge, UK) overnight at 4°C. On the second day, the cells were washed three times with PBS and incubated with the secondary antibody, Alexa Fluor 594 donkey anti-mouse IgG (Molecular Probes, Eugene, OR, USA) (1:500), in the dark for 1 hour. The nucleus of the cells was stained with 4′,6-diamidino-2-phenylindole (DAPI, Molecular Probes) for 10 minutes. A confocal laser scanning microscope was used to examine the stained images. The intensity of CD31 was analyzed using Leica Application Suite X (LASX) (Leica Microsystems CMS GmbH).

## Quantitative real-time polymerase chain reaction

Glucose-treated HUVEC were incubated in EGM with exosome or EGM without exosome for 6, 12, and 24 hours. Total RNA was isolated using TRIzol Reagent (Thermo Fisher Scientific), and each RNA was then reverse transcribed to complementary DNA (cDNA) using the iScript™Select cDNA Synthesis Kit (Bio-Rad, Hercules, CA, USA). qRT-PCR reactions were performed using the KAPA SYBR^®^ FAST qPCR Kit (Fisher Scientific, Loughborough, UK). Glyceraldehyde-3-phosphate dehydrogenase (GAPDH) was used as an internal control. Table 1 shows the PCR primer sequences used.

**Table 1:** Primer sequences for real-time PCR

Gene	Sequences	Temperature (°C)
GATA2	F: 5'-ACTACAGCAGCGGACTCTTC-3'	60
	R: 5'-ACAATTTGCACAACAGGTGCC-3'	
GAPDH	F: 5'-CTCATTTCCTGGTATGACACC-3'	60
	R: 5'-CTTCCTCCTGTGCTCTTGCT-3'	

## Statistical analysis

Each experiment was performed in triplicate and repeated at least three times independently. The results are presented as means ± standard deviation, and the statistical evaluation was performed using PASW Statistics for Windows, Version 18.0. (SPSS Inc., Chicago, USA). A one-way analysis of variance with Tukey’s multiple comparison test was used for all experiments. A p-value < 0.05 was considered as statistical significance.

## Results

### Characterization and internalization of SHED-exosomes

**Figure 1:**
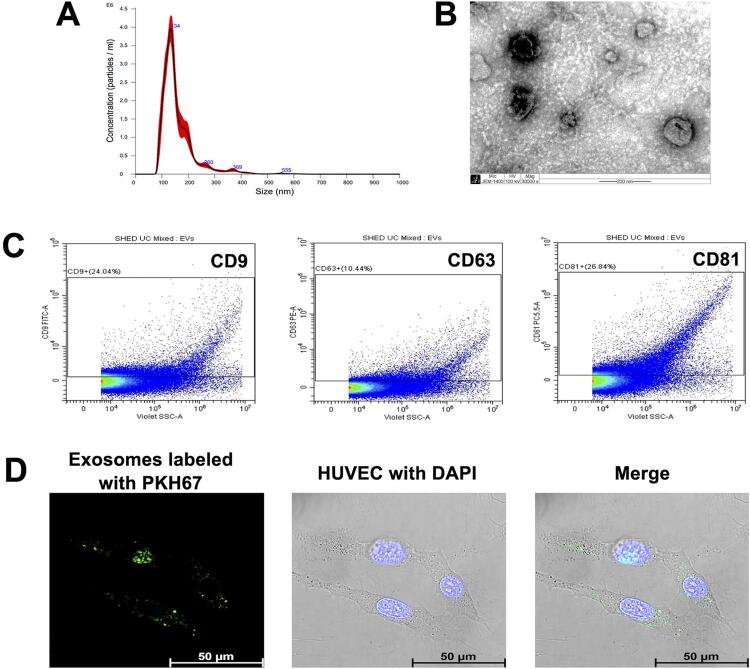
Characterization and internalization of SHED-exosomes. (A) Particle size distribution of SHED-exosomes assessed by nanoparticle tracking analysis (NTA). (B) The morphology of exosomes was observed using a transmission electron microscope (TEM). Scale bar = 200 nm. (C) surface markers of SHED-exosomes were analyzed by flow cytometry and were positive for exosome markers (CD9, CD63, and CD81). (D) Efficient uptake of PKH67-labeled exosomes (green) by HUVEC was detected in four hours. Nuclei were counterstained with DAPI (blue). Scale bars = 50 µm

The diameter of the exosome isolated from SHED CM was accessed by NTA with the camera level set to 14 and the detection threshold to 5 and was shown to range from 60 to 150 nm with a peak of 134 nm ( Figure 1A ). The negative staining and morphology of the SHED-exosomes via a TEM microscope showed the typical cup-shaped particles ( Figure 1B ). Flow cytometry of SHED-exosomes showed the positive expression of the exosome-specific markers CD9, CD63, and CD81 ( Figure 1C ). These findings confirmed that the extracellular vesicles isolated from SHED CM are exosomes. Subsequently, the exosomes were labeled with PKH67 dye to test the interaction between the exosome and HUVEC. The PKH67-labeled exosomes were added to HUVEC. After 4 hours of incubation, the internalization of PKH67-labeled SHED-exosomes was evident by the bright green color in the nucleus and cytoplasm of endothelial cells ( Figure 1D ). The results indicate that the SHED-exosomes with potential biological effects were efficiently taken up by HUVEC.

### Effects of SHED-exosome on the proliferation of hyperglycemia-induced HUVEC

**Figure 2:**
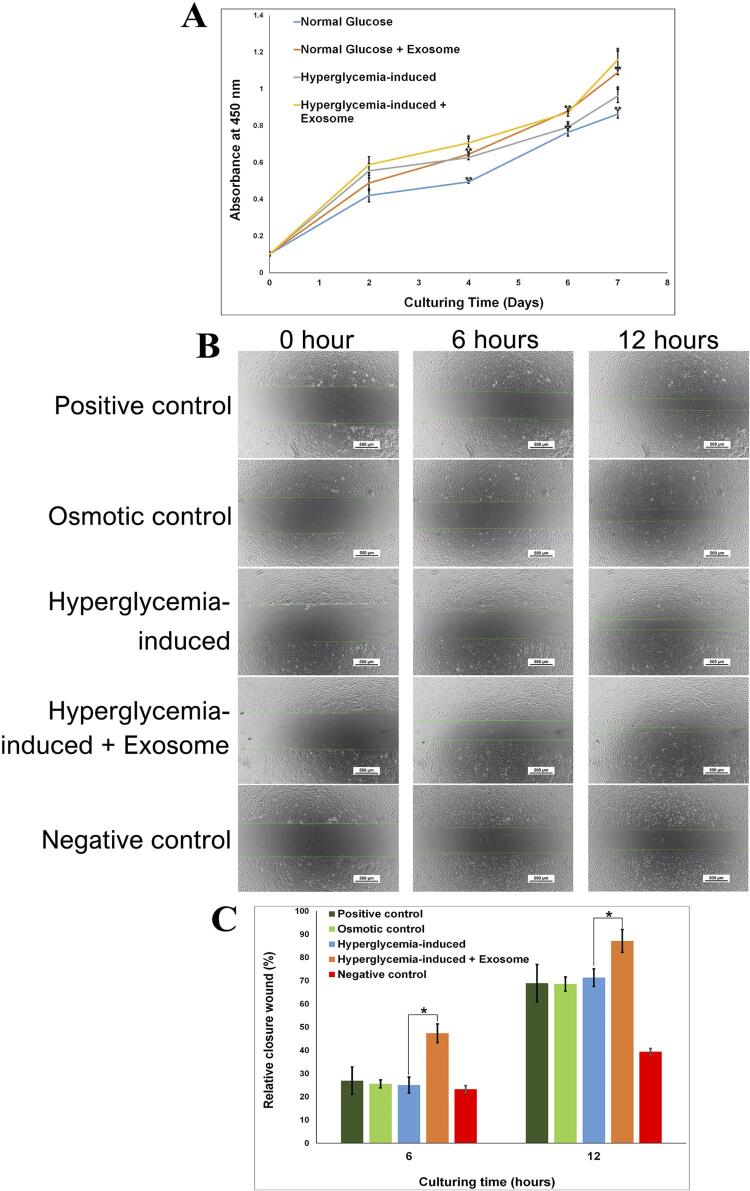
SHED-exosomes induce the growth and migration of hyperglycemia-induced endothelial cells. (A) The line charts represent the mean of the proliferation of HUVEC analyzed using the CCK-8 assay at different time points. **, indicates statistically significant differences between the group of exosomes and no exosome treatment in normal glucose concentration-induced HUVEC at day 4 (p=0.03), day 6 (p=0.03), and day 7 (p=0.02), * indicates statistically significant differences between the group of exosomes and no exosome treatment in high glucose concentration-induced HUVEC at day 4 (p=0.04), day 6 (p=0.03), and day 7 (p=0.02). (B) Analysis of HUVEC migration by *in vitro* scratch assay. Scale bars = 500 µm. (C) Quantification of the rate of scratch closure (%) in B. * indicates statistically significant differences between the group of exosomes and no exosome treatment in high glucose concentration-induced HUVEC at 6 hours (p=0.01) and 12 hours (p=0.03)

The proliferation of HUVEC was a factor related to the initial process of angiogenesis. After incubation with SHED-exosome (10 µg/ml), glucose-treated HUVEC growth was assessed using CCK-8 assays. The number of HUVEC increased continuously from day 0 to day 7 in all groups ( Figure 2A ). HUVEC treated with a high glucose concentration of 25 mM showed higher proliferation than HUVEC treated with 5.5 mM normal glucose concentration. Treatment with SHED-exosome enhanced the growth of both normal and high glucose-treated endothelial cells with statistically significant growth on day 4 (p=0.03 for normal glucose and p=0.04 for high glucose), day 6 (p=0.03 for all treatments), and day 7 (p=0.02 for all treatments) ( Figure 2A ). These findings imply that SHED-exosomes activate the proliferation of hyperglycemia-induced HUVEC cells.

### Effects of SHED-exosome on the migration of high glucose-treated HUVEC

A scratch assay was used to test if SHED-exosome could enhance the migration behavior of high glucose-treated HUVEC as shown in Figure 2B and Figure 2C . The endothelial cells cultured in 5.5 mM normal glucose-treated (positive control), 25 mM mannitol-treated (osmotic control), and 25 mM high glucose-treated (hyperglycemia-induced) exhibited comparable percentages of relative closure wound at 6 hours (26.9±5.8, 25.6±1.7, and 25.1±3.4, respectively) and 12 hours (68.9±8.1, 68.6±3.1, and 71.3±3.8, respectively). Interestingly, hyperglycemia-induced HUVEC cultured in a medium supplemented with 10 µg/ml SHED-exosome showed statistically significant higher relative closure wound values than the high glucose-treated HUVEC at 6 hours (47.4±4.0; p=0.01) and 12 hours (87.1±4.9; p=0.03) ( Figure 2B and Figure 2C ). The HUVEC cultured in DMEM (negative control) showed a lower percentage of relative closure wounds than those other groups (23±1.5 at 6 hours and 39.5±1.3 at 12 hours). These results indicate that SHED-exosome can enhance the migration of hyperglycemic-induced endothelial cells.

### Effect of SHED-exosome on the tube formation

**Figure 3:**
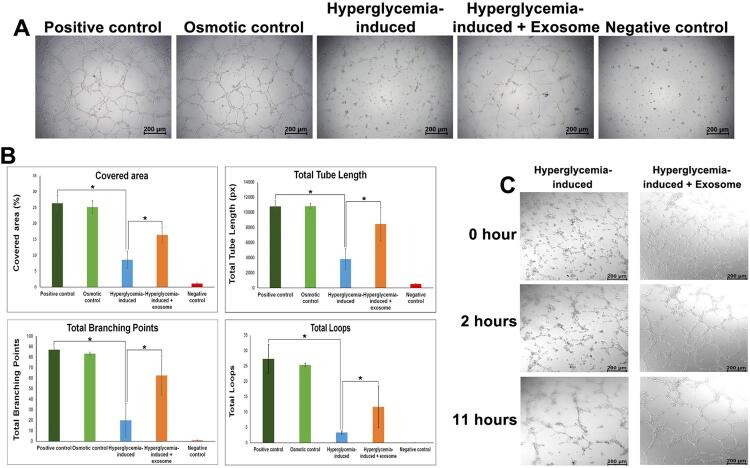
SHED-derived-exosome promotes tube formation in high glucose concentration-treated HUVEC. (A) Capillary-like network structures in 6 hours were examined using Matrigel tube formation. Scale bars = 200 µm. (B) Quantification of tube length, number of tube branching points, covered area, and number of loops in the network structures of HUVEC in A were analyzed using WimTube image analysis software. * indicates a statistically significant difference between the group of high glucose concentration-induced and positive control (p<0.001) and between the group of exosomes and no exosome treatment in high glucose concentration-induced (p=0.03). (C) Representative images from the live tube formation were captured of uncontrolled blood glucose concentration-treated HUVEC with and without SHED-exosome treatment at 0, 2, and 11 hours. Scale bars = 200 µm

Tube formation experiments on Matrigel-coated wells were conducted to study the proangiogenic effects of SHED-exosome on glucose-treated HUVEC, as shown in Figure 3 . As expected, at 6 hours HUVEC formed capillary-like networks in positive and osmotic control groups ( Figure 3A ), indicating no osmotic effect on the tube formation. No complex tubular structures in the HUVEC in negative control were observed. On the other hand, hyperglycemia-induced HUVEC barely formed any mesh-like structures, and most of the tubes failed to connect with each other, presenting a discontinued appearance. Interestingly, hyperglycemia-induced HUVEC cultured in a medium supplemented with SHED-exosome formed noticeable mesh-like structures ( Figure 3A ). The high glucose-treated HUVEC had the thinnest tubes, some of which were even discontinued in some areas. In contrast, the high glucose-treated endothelial cells supplemented with exosomes exhibited similar tube branch thickness compared to the positive control. In addition, mesh-like structures were formed more in the high glucose concentration-treated HUVEC-supplemented exosomes group than in the hyperglycemia-induced group ( Figure 3A ).

The covered area, total tube length, total branching points, and total loops of each group were then quantified using WimTube image analysis ( Figure 3B ). Mesh-like structures in the hyperglycemia-induced group were significantly lower in all aspects (p<0.001) compared to the positive control group, confirming increased glucose concentration’s detrimental effects on angiogenesis. However, mesh-like structures in the HUVEC-supplemented SHED-exosomes in the high glucose concentration-treated group were substantially higher than those in the high glucose-treated group (p=0.03; Figure 3B ).

A 17-hour-long video of HUVEC tube formation was recorded to observe tube differences between hyperglycemia-induced HUVEC treated with and without SHED-exosomes (Video link at shorturl.at/bjwEZ). Both groups started with similarly dispersed HUVEC with no tubes formed ( Figure 3C and video link at shorturl.at/esKSX). Over time, cells began to migrate toward each other. Within the first two hours, noticeable tubes were observed for both groups. A greater organized network formation, loop formation, branching points, thicker tubes, and a faster rate of tube formation were observed in the exosomes-treated endothelial cells group. In contrast, the high glucose-treated HUVEC formed more disorganized networks, fewer loops and branching points, thinner tubes, and tube formation at a slower rate ( Figure 3C and video link at shorturl.at/bjwEZ). After 11 hours, while the tubes of the HUVEC treated with exosomes maintained the thickness and had complex networks, the tubes of the HUVEC that were not treated with exosomes began to gradually disconnect ( Figure 3C and video link at shorturl.at/bjwEZ). Together, these findings imply that SHED-exosomes induce the tube formation characteristics of high glucose concentration-treated HUVEC.

### Effect of SHED-exosome on specific angiogenesis genes and protein expression

**Figure 4:**
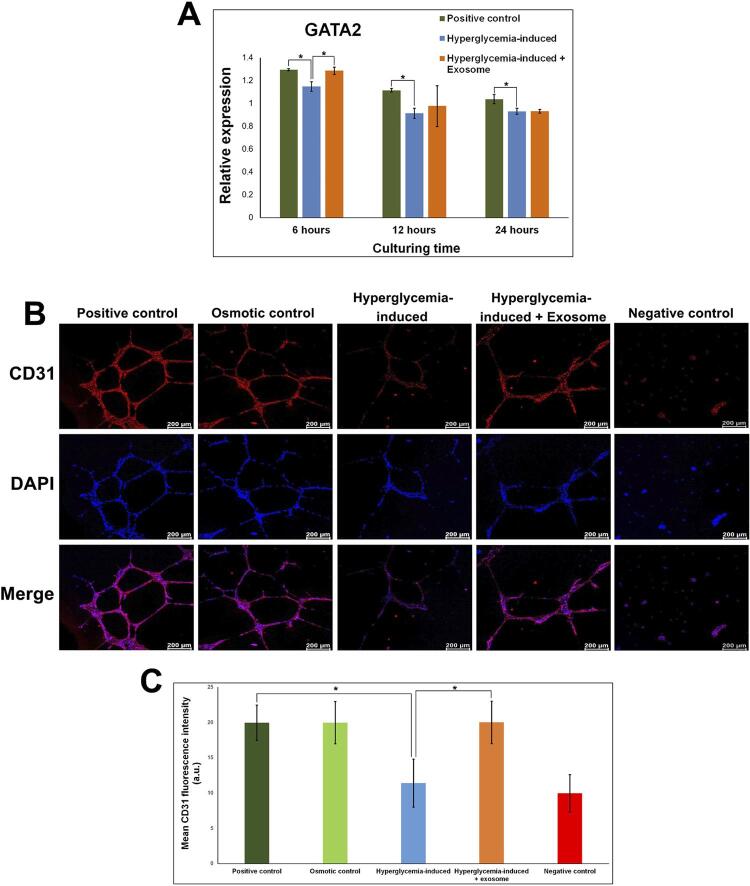
SHED-exosomes enhance angiogenesis gene and protein expression of hyperglycemia-induced HUVEC. (A) Quantitative real-time PCR relative GATA2 gene expression level of cells in various groups at hours 6, 12, and 24. The bar charts represent the mean of gene expression relative to GAPDH expression, with the error bar showing the standard deviation of gene expression. * indicates a statistical difference between the groups (p=0.03). (B) Immunofluorescence images of tube network structures in five sample groups in 12 hours; the upper panel shows CD31 expression (red), the middle panel shows DAPI (blue), and the lower panel shows a merge between CD31 and DAPI. Scale bars = 200 µm. (C) Graph represents the mean fluorescence intensity of CD31 for positive control, osmotic control, hyperglycemia-induced, hyperglycemia-induced + exosome, and negative control groups. * indicates a statistical difference between the groups (p=0.03)

RT-PCR was used to quantify the expression of the GATA2 gene in various groups, as shown in Figure 4A . GATA2 controls endothelial programming during the endothelial-to-hematopoietic transition. GATA2 mRNA levels in hyperglycemia-induced HUVEC were significantly lower than HUVEC treated with normal glucose concentrations (positive control) at 6, 12, and 24 hours (p=0.03 in all times). However, high glucose-treated HUVEC cultured with SHED-exosomes showed an increase in GATA2 gene expression from 6 hours to 24 hours compared to untreated exosomes with statistical significance at 6 hours (p=0.03) ( Figure 4A ).

In order to qualitatively examine angiogenesis in the experimental groups, immunofluorescence staining with CD31 was performed. The tube-like structures from the tube formation assay were stained and observed under a confocal microscope for 12 hours, as shown in Figure 4B . Although at varying degrees of intensity, all groups showed positively stained DAPI and were likewise positively stained for the CD31 protein. Both positive and osmotic control showed interconnecting tube networks of elongated CD31-positive cells. On the other hand, the negative control exhibited clumping of cells without tube-like structure formation and a visible lower intensity in CD31 staining compared to the positive control ( Figure 4B and Figure 4C ). Most evidently, the intensity of CD31 fluorescence in the high glucose concentration-treated group was significantly lower than the positive control (p=0.03), having a similar intensity to the negative control. Compared to the group treated with high glucose, the intensity of the CD31 positively stained in HUVEC treated with SHED-exosome was significantly higher (p=0.03: Figure 4B and Figure 4C ). The results indicate that SHED-exosomes help to recover the GATA2 gene and CD31 glycoprotein expression angiogenesis in hyperglycemic-induced HUVEC.

## Discussion

This study was the first to observe that SHED-derived exosomes enhance the angiogenesis behavior of endothelial cells induced by hyperglycemia. Diabetes, associated with hyperglycemia or elevated blood glucose levels, is linked to periodontitis.^3^ Periodontitis treatment aims to regenerate damaged tissue, but diabetic patients’ regeneration worsens due to poor angiogenesis.^7^ Meanwhile, we showed that 4 days of treatment with 25 mM uncontrolled blood glucose concentration significantly reduced the angiogenic potential of HUVEC. For instance, the high glucose-treated HUVEC exhibited significantly increased apoptotic cell number via up-regulation of the Bax/Bcl-2 ratio and activation of caspase-3 involved with the mitochondrial apoptotic pathway.^32^ High glucose significantly reduced the anti-apoptotic VEGF expression in HUVEC at mRNA and protein levels by inhibiting p42/44 MAPK.^32^ Moreover, high glucose treatment also reduces the expression of sirtuin1 and forkhead box O_3_, thus resulting in increased apoptosis in HUVEC.^33^

Exosomes are special EVs that function as important paracrine mediators. MSC-derived exosomes contain bioactive molecules, including proteins, lipids, signaling molecules, miRNAs, and mRNAs.^34^ The exosomes act as nanocarriers to transfer bioactive molecules from parent cells to recipient cells and modulate their functions. The SHED is selected as seeded cells for exosome production rather than adult dental pulp stem cells because of their higher proliferation, differentiation capacities, and proangiogenic effect.^35^ Furthermore, the underlying therapeutic mechanism of SHED-derived exosomes toward hyperglycemic conditioned HUVEC was not explored. In this study, SHED-derived exosomes were cup-shaped, and the diameters of the nanoparticles ranged from 60 nm to 150 nm, with a peak at 134 nm. Flow cytometry analysis confirmed the presence of CD9, CD63, and CD81, which aligns with exosome characterization recommendations of the Minimal Information for Studies of Extracellular Vesicles 2018 (MISEV2018) and other studies.^23 , 25 , 36 - 38^ Finally, exosome internalization into the HUVEC was demonstrated by PKH-67 labeling, and the labeled exosomes were located in the cytoplasm and nucleus. Other studies also found that exosomes accumulate inside the cells even with different labeling techniques.^23 , 25 , 36 - 38^ These exosomes potentially produced biological effects on endothelial cells by means of paracrine function.

We conducted experiments to prove that 10 µg/ml SHED-exosomes enhance the growth and migration of hyperglycemia-induced endothelial cells. MSC-derived exosomes can transport diverse signaling factors to recipient cells to regulate cell proliferation and/or angiogenesis and overcome the disadvantages of direct MSC transplantation.^39 - 41^ Previous studies showed that 30 µg/ml hypoxic-preconditioned SHED and 5, 10, and 30 µg/ml normally cultured SHED-derived exosomes can augment the proliferation, migration, and tube formation of HUVEC.^25 , 42^ In contrast, a recent report by Liu P et al. found that 30 µg/ml SHED-exosome suppressed cell growth and significantly induced apoptosis of endothelial cells.^37^ Additionally, migration assays also showed significant suppression of the migration and invasion of endothelial cells after treatment with SHED-exosomes. This difference possibly occurred because of different donor cells, different intervals, concentration of exosomes used, and culturing methods of SHED, resulting in exosomal cargoes with different components, such as different miRNAs.^37^ The evidence shows that the contents of exosomes are dynamic and largely depend on cellular origin and physiological status.^43^ The molecular composition not only depends on the cell type of origin but also on the microenvironment, which includes mechanical properties, topography, and the presence of activating biochemical stimuli that regulate the protein cargo of the secreted exosomes. The induction of endothelial cell proliferation and migration may also contribute to the activation of angiogenesis.

In our study, HUVEC cultured with SHED-exosomes formed abundant visible tube-like structures, especially compared to the high glucose-treated HUVEC, which formed minimal tubular structures. These findings are supported by numerous research that shows SHED-derived exosomes exhibit proangiogenic properties *in vitro* and *in vivo* experiments.^22 - 23 , 25 , 35^ The evidence shows that the VEGF expression of HUVEC or MSCs treated with SHED-exosomes is significantly upregulated.^22 - 23 , 25 , 35^ The activities of VEGF extend beyond the vascular system, which plays a role in normal physiological functions such as bone formation, hematopoiesis, wound healing, and development. When HUVEC are cultured with SHED-exosomes, total tube length increases by 1.5 times, and the number of junctions increases by two times compared to the control group, according to an earlier study that confirmed similar results for the tube formation assay.^25^ In contrast, some research showed that SHED-exosomes could inhibit angiogenesis *in vitro* .^37^

SHED-derived exosomes promote angiogenic gene expression, including VEGFa, KDR, FGF2, angiogenin, and PDGF, suggesting their proangiogenic potential.^20 , 23 , 25 , 34^ With a common origin, hemangioblasts, blood, and endothelial cells share the expression of a number of different genes and several transcription factors involved in the commitment and differentiation of hemangioblasts to hematopoietic and endothelial cells.^44^ GATA2 is a transcription factor expressed in early progenitors with potential to generate both hematopoietic and endothelial cells. GATA2 regulates the promoting activity of several endothelium-specific genes, such as platelet endothelial cell adhesion molecule-1 (PECAM-1) and endothelin-1. Furthermore, GATA2 regulates the expression of VEGFR-2 during both vascular development and angiogenesis, the process by which endothelial cells form new blood vessels from an existing vascular network.^44 - 46^ In our study, the proangiogenic effects of SHED-exosomes were demonstrated through an increase in the gene expression of GATA 2; a transcription factor expressed in early progenitors with the potential to generate both hematopoietic cells and endothelial cells, in glucose-treated HUVEC using RT-PCR.

Furthermore, the CD31 or PECAM-1 interaction supports robust endothelial cell adhesion, which is present at the intercellular junction.^47^ In this study, the CD31 fluorescence intensity in the group treated with high glucose concentrations was much lower than that in the positive control. It was comparable to the negative control, confirming the hypothesis that glucose reduces endothelial cell angiogenesis. Interestingly, CD31 protein in the high glucose concentration-treated HUVEC supplemented with exosomes returned to high expression with a fluorescence intensity similar to that of the positive group. In contrast to the SHED-only transplanted group and control group, the previous study showed that after *in vivo* implantation of SHED aggregate and SHED aggregate exosomes, regenerated tissue showed the highest CD31 intensity in the sample group containing SHED aggregate exosome.^25^ Using SHED aggregate exosomes promoted regeneration and angiogenesis, as immunofluorescence staining showed newly formed CD31 positive vessels, thus confirming the angiogenic process.^23^ It further supports our results that the use of SHED-derived-exosomes promotes angiogenesis.

## Conclusions

This study showed that SHED-exosome enhances vascular endothelial cell function suppressed by hyperglycemia. They included increased cell proliferation, cell migration, capillary-like tube formation, GATA2 gene expression, and CD31 protein expression. These results suggest that the administration of SHED-exosome improves vascular endothelial cell dysfunction in diabetic patients. The administration of SHED-exosome may improve periodontal treatment in diabetic patients because periodontal patients with diabetes respond poorly to conventional periodontal therapy. Exosomes have excellent biocompatibility and high stability and are expected to be excellent therapeutic agents. The results of this study showing that SHED-exosome improves vascular endothelial cell function in hyperglycemic conditions may provide a new treatment strategy for periodontal patients with diabetes mellitus.
