# ERRATUM

**DOI:** 10.1590/1678-7757-2023er002

**Published:** 2023-06-12

Due to a publishing error the article: “Stem cell-derived exosomes from human exfoliated deciduous teeth promote angiogenesis in hyperglycemic-induced human umbilical vein endothelial cells”, published at Journal of Applied Oral Science 31(e-20220427):1-13. doi: 10.1590/1678-7757-2022-0427 was printed with the following error:

Where it reads:

Acknowledgments

The authors thank Natthapatt Sooppapipatt, Pitchaporn Teacharushatakit, Wifada Powattanasuk, and Chareerut Phruksaniyom for their technical assistance. Moreover, the authors thank Enago (www) for the English language review.

The sentence should read:

Acknowledgments

The authors thank Natthapatt Sooppapipatt, Pitchaporn Teacharushatakit, Wifada Powattanasuk, and Chareerut Phruksaniyom for their technical assistance. Moreover, the authors thank Enago (www) for the English language review.

This research work is supported by Mahidol University (Basic Research Fund: fiscal year 2022).

